# Computed tomography findings and surgical outcomes in acute mesenteric ischemia: a retrospective single-center cohort study

**DOI:** 10.3389/fsurg.2026.1824037

**Published:** 2026-06-24

**Authors:** Andrea Cavallaro, Antonio Zanghì, Alessandro Cappellani, Francesco Leonforte, Antonio Mistretta, Mariacristina Micalizzi, Paolo Di Mattia, Massimiliano Veroux, Kenya Tiralongo

**Affiliations:** 1 General Surgery III, Department of General Surgery and Medical-Surgical Specialties, University of Catania, Catania, Italy; 2 Department of General Surgery and Medical-Surgical Specialties, Chief ChiSMaCoTA Research Center, AOU Policlinico “G. Rodolico – San Marco”, Catania, Italy; 3 Department of Integrated Hygiene, Organizational, and Service Activities (Structural Department), Health Management, University Hospital Polyclinic “G. Rodolico-San Marco”, Catania, Italy; 4 Department of Medical and Surgical Sciences and Advanced Technologies, University of Catania, AOU Policlinico “G. Rodolico – San Marco”, Catania, Italy; 5 Department of Vascular Surgery, Helios Klinikum München West, Munich, Germany; 6 General Surgery, Umberto I Hospital, Department of Medicine and Surgery, Kore University, Enna, Italy; 7 General Surgery III, Organ Transplant Unit, Department of Medical and Surgical Sciences and Advanced Technologies, University of Catania, AOU Policlinico “G. Rodolico – San Marco”, Catania, Italy

**Keywords:** 30-day mortality, acute mesenteric ischemia, bowel necrosis, computed tomography, emergency surgery, non-occlusive mesenteric ischemia, pneumatosis intestinalis, radiological predictors

## Abstract

**Background:**

Acute mesenteric ischemia (AMI) is a time-dependent condition associated with high mortality, frequently related to diagnostic delay and progression to irreversible bowel necrosis. Computed tomography (CT) plays a pivotal role not only in diagnosis but also in guiding therapeutic decision-making. However, the prognostic role of individual CT findings and their relationship with irreversible ischemic injury remain incompletely defined. This study evaluated predefined CT findings, AMI subtype, treatment pathways, intraoperative infarction or necrosis, and mortality in a real-world cohort of patients with CT-confirmed AMI.

**Methods:**

A retrospective single-center cohort study was conducted including 102 adult patients with CT-confirmed AMI admitted between January 2018 and February 2026. Patients were classified by AMI subtype as arterial occlusive AMI, venous AMI, non-occlusive mesenteric ischemia (NOMI), or secondary/mechanical ischemia. Eight predefined CT findings were analyzed: pneumatosis intestinalis, porto-mesenteric venous gas, bowel wall thickening, colitis/ileitis, occlusion/volvulus/intussusception, free intraperitoneal air, free peritoneal fluid, and vascular suffering or bowel wall hypoenhancement. Radiological diagnostic certainty was analyzed separately as a synthetic imaging variable. The primary endpoint was intraoperative evidence of bowel infarction, necrosis, or irreversible ischemia among surgically explored patients. Secondary endpoints included clinically significant ischemia, bowel resection, invasive treatment, in-hospital mortality, and 30-day mortality. Univariate associations, diagnostic performance measures, ROC/AUC analysis, and parsimonious multivariable logistic regression models for mortality were performed.

**Results:**

Arterial occlusive AMI was the most frequent AMI subtype (59/102, 57.8%), followed by NOMI (23/102, 22.5%), secondary/mechanical ischemia (15/102, 14.7%), and venous AMI (5/102, 4.9%). In-hospital mortality was 41.2% (42/102), while 30-day mortality was 46.5% (47/101), including five deaths after discharge. The primary endpoint was assessable in 65 patients; intraoperative infarction or necrosis was present in 53 (81.5%). No individual CT finding showed strong standalone discriminatory performance for intraoperative infarction or necrosis. Pneumatosis showed the strongest descriptive performance for the primary endpoint (OR: 2.72, 95% CI: 0.76–9.80; *p* = 0.188; AUC 0.62). Pneumatosis showed an exploratory association with 30-day mortality (OR: 2.42, 95% CI: 1.08–5.43; *p* = 0.045). In the multivariable model for 30-day mortality, acute kidney injury was independently associated with death (adjusted OR: 9.26, 95% CI: 2.40–35.74; *p* = 0.001), while non-surgical admission showed a borderline association after adjustment (adjusted OR: 2.70, 95% CI: 0.94–7.71; *p* = 0.065). The model AUC was 0.82.

**Conclusions:**

In this heterogeneous AMI cohort, individual CT findings were clinically meaningful but showed limited standalone discriminatory performance for surgically confirmed infarction or necrosis. Pneumatosis showed an exploratory association with 30-day mortality, while porto-mesenteric venous gas and free air were specific but infrequent. CT remains essential for diagnosis, subtype classification, and treatment planning, but CT findings should be interpreted within an integrated clinical and multidisciplinary framework. The exploratory CT ischemic burden score should be considered descriptive and not a validated triage or prognostic tool.

## Introduction

Acute mesenteric ischemia (AMI) represents one of the most severe abdominal emergencies, characterized by abrupt or progressive impairment of intestinal blood supply that may rapidly evolve from reversible mucosal ischemia to transmural infarction, sepsis, multiorgan failure, and death. Although AMI is relatively uncommon compared with other causes of acute abdomen, estimated at approximately 0.1%–0.2% of emergency surgical admissions, it remains associated with high mortality, ranging from 40% to over 50%, particularly in elderly and frail patients and in those with delayed diagnosis or advanced systemic deterioration (1–3).

AMI encompasses a heterogeneous group of pathophysiological conditions associated with a progressive spectrum of intestinal injury, ranging from reversible ischemia to irreversible transmural necrosis, with major implications for prognosis and management. Arterial occlusive AMI is commonly caused by embolic or thrombotic obstruction of the superior mesenteric artery. Venous AMI is related to mesenteric venous thrombosis and venous congestion. Non-occlusive mesenteric ischemia (NOMI) occurs in the absence of major vessel occlusion and is often associated with low-flow states, vasoconstriction, shock, or critical illness. Secondary or mechanical ischemia may occur in the setting of volvulus, strangulated obstruction, intussusception, or other mechanical causes compromising intestinal perfusion. These subtypes differ in imaging appearance, therapeutic strategy, and prognosis.

Clinical diagnosis remains challenging because symptoms are frequently non-specific, particularly in elderly or critically ill patients. Laboratory biomarkers may support severity assessment but are not sufficiently reliable to exclude or confirm early ischemia (1–4). Contrast-enhanced CT is therefore central to the diagnosis of AMI, allowing assessment of vascular anatomy, bowel wall abnormalities, extraluminal complications, and potential disease extent.

Typical CT findings include reduced or absent bowel wall enhancement, bowel wall thickening or thinning, pneumatosis intestinalis, porto-mesenteric venous gas, free intraperitoneal air, free peritoneal fluid, mesenteric fat stranding, arterial or venous occlusion, and signs of mechanical obstruction or strangulation. However, the prognostic value of individual CT findings remains complex. Some signs, such as pneumatosis and porto-mesenteric venous gas, are often considered markers of advanced ischemia, but their meaning depends on AMI subtype, associated enhancement abnormalities, systemic condition, and timing of imaging (4–7). The aim of this study was to evaluate CT findings and clinical outcomes in patients with AMI in a real-world single-center cohort. The analysis focused on predefined CT findings, AMI subtype classification, intraoperative evidence of bowel infarction or necrosis, and 30-day mortality. A simplified exploratory CT ischemic burden score based exclusively on eight radiological findings available in the dataset was also assessed.

## Materials and methods

### Study design and population

A retrospective, single-center cohort study was conducted at the University Hospital “G. Rodolico – San Marco” in Catania, Italy. The study included adult patients admitted between January 2018 and February 2026 with computed tomography-confirmed acute mesenteric ischemia. The aim of the study was to evaluate the association between predefined CT findings, AMI subtype, treatment strategy, intraoperative evidence of irreversible ischemic injury, and clinical outcomes. Adult patients (≥18 years) presenting with CT and clinical findings suggestive of intestinal ischemia were eligible for inclusion. Radiological criteria included one or more findings suggestive of intestinal ischemia or ischemia-related complications, including pneumatosis intestinalis, porto-mesenteric venous gas, abnormal bowel wall enhancement or vascular suffering, bowel wall thickening, colitis or ileitis, free intraperitoneal air, free peritoneal fluid, mesenteric arterial or venous occlusion, volvulus, obstruction, intussusception, or other mechanical causes of bowel ischemia.

Patients with insufficient clinical or radiological documentation to allow meaningful classification were excluded. The final study cohort included 102 patients.

All patients were classified according to AMI subtype. Four categories were used: arterial occlusive AMI, venous AMI, non-occlusive mesenteric ischemia, and secondary or mechanical ischemia. Secondary/mechanical ischemia included cases related to volvulus, bowel obstruction, strangulation, intussusception, or other mechanical conditions leading to ischemic intestinal injury. AMI subtype classification was based on CT findings, clinical presentation, operative findings when available, and review of the medical records. AMI is a heterogeneous disease entity, and different subtypes may differ in pathophysiology, CT appearance, therapeutic management, and prognosis.

### Radiological assessment and exploratory CT ischemic burden score

All CT examinations were reviewed, according to a structured radiological framework (4–6), by radiologists and expert emergency surgeons. The analysis focused on eight predefined CT findings that were available in the dataset and were considered potentially relevant to intestinal ischemia severity.

The eight CT findings were pneumatosis intestinalis, porto-mesenteric venous gas, bowel wall thickening, colitis or ileitis, occlusion/volvulus/intussusception, free intraperitoneal air, free peritoneal fluid, and vascular suffering or bowel wall hypoenhancement. Each radiological feature was coded as a binary variable indicating presence or absence of the finding.

The composite variable “occlusion/volvulus/intussusception” was used to identify CT evidence of a mechanical or obstructive mechanism potentially associated with ischemic progression. It included bowel obstruction with a transition point, closed-loop configuration, volvulus with twisting of the mesentery or mesenteric vessels, intussusception, strangulation features, or other obstructive/mechanical findings judged to contribute to bowel ischemia.

The variable “colitis/ileitis” referred to an overall CT pattern of colonic or ileal involvement rather than isolated wall thickening. It included segmental or diffuse bowel wall thickening or mural edema associated with abnormal contrast enhancement, including mucosal hyperenhancement or reduced mural enhancement, and/or pericolic or peri-ileal fat stranding or adjacent fluid, when interpreted as compatible with ischemic colitis or ileitis.

Pneumatosis intestinalis and porto-mesenteric venous gas were considered markers of advanced bowel wall injury and gas dissection into the bowel wall or venous system. Vascular suffering or bowel wall hypoenhancement was considered a marker of impaired perfusion. Free intraperitoneal air was considered a possible marker of perforation or advanced transmural injury. Free peritoneal fluid was considered a non-specific but potentially relevant marker of intra-abdominal inflammatory or ischemic involvement. Radiological diagnostic certainty was also recorded as a separate synthetic imaging variable. This variable identified patients with definite or highly probable radiological ischemia, as opposed to unlikely or doubtful ischemia. Radiological certainty was not treated as an individual CT sign, but as an overall radiological interpretation based on the complete CT pattern.

An exploratory CT ischemic burden score was calculated by assigning one point for each of the eight predefined CT findings. The score ranged from 0 to 8, with higher values reflecting a greater cumulative burden of ischemia-related CT abnormalities. The score was not intended as a validated prognostic model, diagnostic score, or triage tool. It was used only as an exploratory descriptive measure of radiological disease burden. No therapeutic threshold was proposed, and the score was not used to guide treatment decisions.

### Clinical management and therapeutic strategies

Treatment strategy was classified according to the actual management performed during the index hospitalization. Patients were categorized as receiving non-invasive/conservative treatment, surgical treatment, interventional radiology/endovascular treatment, or combined surgical and interventional treatment. Surgical treatment included exploratory laparotomy or laparoscopy, bowel resection, stoma creation, second-look surgery, or other operative procedures performed for suspected or confirmed bowel ischemia. Interventional radiology or endovascular treatment included procedures aimed at restoring or improving mesenteric perfusion or treating vascular occlusion, when performed. Combined treatment referred to patients who underwent both surgical and interventional/endovascular procedures during the same clinical episode. Because treatment allocation was not randomized and was strongly influenced by AMI subtype, radiological findings, clinical severity, anatomical feasibility, hemodynamic status, and surgical judgment, treatment-related analyses were considered descriptive and exploratory. No causal inference regarding the efficacy of invasive, surgical, or endovascular treatment was attempted.

### Clinical variables and outcomes

Clinical data were extracted from electronic hospital records and included demographic characteristics, pre-existing comorbidities, and acute complications. Variables included age, sex, AMI subtype, initial admitting department, treatment strategy, surgery, interventional radiology/endovascular treatment, bowel resection, acute kidney injury, diabetes mellitus, pulmonary disease, cardiac disease, neurological disease, oncohematologic disease, sepsis, in-hospital mortality, and 30-day mortality.

The initial admitting department was classified as surgical or non-surgical. This variable was considered an organizational and clinical pathway variable. Because admission to a non-surgical department may reflect atypical presentation, greater systemic illness, frailty, respiratory or cardiovascular instability, or diagnostic uncertainty, associations involving this variable were interpreted cautiously and not as evidence of causality.

The primary endpoint was defined only in patients who underwent surgical exploration with direct assessment of bowel viability. Patients were classified as positive when bowel infarction, necrosis, non-viable bowel, or irreversible ischemia was documented intraoperatively, regardless of whether bowel resection was performed. This included patients in whom ischemic injury was judged intraoperatively to be too extensive for meaningful or technically feasible resection. Patients were classified as negative when operative assessment documented viable bowel without infarction, necrosis, or irreversible ischemic injury. Patients without direct intraoperative assessment of bowel viability were considered not assessable for this endpoint.

Secondary endpoints included clinically significant intestinal ischemia, invasive treatment, bowel resection, in-hospital mortality, 30-day mortality, and non-surgical admission. Clinically significant ischemia was defined as a broader clinical endpoint including intraoperative infarction or necrosis, bowel resection for ischemia or necrosis, histological confirmation when available, or strong clinical-radiological evidence of irreversible ischemia in patients who were not operated on because of extensive disease, prohibitive clinical condition, or rapid deterioration. Death alone was not considered sufficient to define clinically significant ischemia unless supported by strong clinical and radiological evidence of irreversible ischemic injury.

Bowel resection was analyzed as a therapeutic endpoint among patients in whom surgical evaluation was available. In-hospital mortality was defined as death occurring during the index hospitalization. Thirty-day mortality was defined as death occurring within 30 days of the AMI episode, including both in-hospital deaths and documented deaths after discharge, including patients discharged voluntarily who died at home within 30 days.

### Statistical analysis

Statistical analysis was performed according to a predefined exploratory analysis plan using Stata software version 13 (StataCorp, College Station, TX, USA).

Continuous variables were reported as mean ± standard deviation, while categorical variables were reported as absolute frequencies and percentages. The primary analysis evaluated the association between radiological variables and intraoperative evidence of bowel infarction or necrosis. The radiological variables included the eight predefined CT findings and radiological diagnostic certainty. The exploratory CT ischemic burden score was analyzed separately as a continuous score.

For each radiological variable and each outcome, univariate associations were assessed using Fisher's exact test or the chi-square test, as appropriate. Odds ratios with 95% confidence intervals, *p*-values, sensitivity, specificity, positive predictive value, negative predictive value, area under the receiver operating characteristic curve, and phi coefficient were calculated. The phi coefficient was used as a binary correlation coefficient between each radiological variable and the corresponding binary outcome.

The same analytical framework was applied to intraoperative infarction or necrosis, invasive treatment, clinically significant ischemia, bowel resection, in-hospital mortality, 30-day mortality, and non-surgical admission. Associations involving invasive treatment and non-surgical admission were considered exploratory and descriptive, because both variables may be influenced by clinical decision-making and care pathway organization. The exploratory CT ischemic burden score was evaluated against the main study outcomes using logistic regression and ROC curve analysis. Because the score was not externally validated and was intended only as a descriptive measure of radiological burden, its performance was interpreted cautiously.

Multivariable logistic regression models were built for mortality outcomes. The main prognostic model used 30-day mortality as the dependent variable. A secondary model was built for in-hospital mortality. Candidate clinical variables included age, sex, non-surgical admission, acute kidney injury, diabetes mellitus, pulmonary disease, cardiac disease, neurological disease, oncohematologic disease, and sepsis. All clinical variables were first evaluated in univariate analysis. Multivariable models were then kept deliberately parsimonious to avoid overfitting, given the sample size and number of events.

The final mortality models included age, non-surgical admission, acute kidney injury, sepsis, and pulmonary disease. These variables were selected *a priori* on clinical grounds rather than through an automated univariable *p*-value threshold. Selection was based on their relevance to systemic severity, biological plausibility, and their importance in evaluating whether the association between non-surgical admission and mortality persisted after adjustment for acute clinical deterioration. Univariable analyses were used descriptively to characterize crude associations, but no fixed *p*-value cut-off was applied for entry into the multivariable models. This strategy was chosen to avoid overfitting and data-driven variable selection in a relatively small cohort.

Treatment variables, bowel resection, intraoperative infarction/necrosis, and clinically significant ischemia were not included in the main mortality model because they may represent intermediate events or consequences of disease severity and management decisions rather than baseline predictors.

All analyses were considered exploratory and hypothesis-generating. A two-sided *p*-value <0.05 was considered statistically significant for descriptive purposes. Given the retrospective design, the absence of a prospectively registered statistical protocol, the limited number of patients in some subgroups, and the number of radiological variables evaluated across multiple outcomes, no formal correction for multiple comparisons was applied. Therefore, *p*-values were interpreted as descriptive measures of the strength of association rather than as confirmatory hypothesis tests. Results were interpreted according to clinical relevance, biological plausibility, effect size magnitude, and confidence interval width, and require validation in independent prospective cohorts before being used to inform clinical decision-making or treatment algorithms.

### Ethical considerations

This retrospective observational study was conducted in accordance with the ethical standards of the 1964 Declaration of Helsinki and its later amendments or comparable ethical standards. The study did not involve primary research, experimental interventions, or the implementation of new clinical protocols. All data analyzed were collected as part of routine diagnostic and therapeutic management. When non-routine medical procedures, including surgical interventions, were performed, written informed consent was obtained from patients or their legal guardians and included authorization for the use of anonymized data for research and scientific purposes. According to local regulations, the institutional ethics board does not require formal approval for retrospective analyses of aggregated and anonymized data derived from routinely collected clinical records.

## Results

### Patient characteristics

A total of 102 patients with CT-confirmed acute mesenteric ischemia were included in the final analysis (Table 1). The mean age was 73.0 ± 15.9 years, and 55 patients were male, corresponding to 53.9% of the cohort. AMI subtype classification showed that arterial occlusive AMI was the most frequent subtype, occurring in 59/102 (57.8%). NOMI was identified in 23/102 (22.5%), secondary or mechanical ischemia in 15/102 (14.7%), and venous AMI in 5/102 (4.9%). This revised distribution highlights the heterogeneity of the study population and the need to interpret CT findings and outcomes according to the underlying AMI mechanism.

**Table 1:** Baseline characteristics, AMI subtypes, treatment strategies, comorbidities, and clinical outcomes of the study cohort.

Characteristic	Value
Total patients	102
Age, years, mean ± SD	73.0 ± 15.9
Male sex	55/102 (53.9%)
Length of stay, days, mean ± SD	11.6 ± 10.9
AMI subtype: arterial occlusive	59/102 (57.8%)
AMI subtype: NOMI	23/102 (22.5%)
AMI subtype: secondary/mechanical	15/102 (14.7%)
AMI subtype: venous	5/102 (4.9%)
Surgery performed	64/102 (62.7%)
Interventional radiology performed	10/102 (9.8%)
Invasive treatment	72/102 (70.6%)
Bowel resection (among assessable)	36/64 (56.2%)
Intraoperative infarction/necrosis (among assessable)	53/65 (81.5%)
Clinically significant ischemia	75/102 (73.5%)
In-hospital mortality	42/102 (41.2%)
30-day mortality	47/101 (46.5%)
Non-surgical admission	54/102 (52.9%)
AKI/IRA	23/102 (22.5%)
Diabetes	16/102 (15.7%)
Pulmonary disease	30/102 (29.4%)
Cardiac disease	36/102 (35.3%)
Neurological disease	10/102 (9.8%)
Oncohematologic disease	21/102 (20.6%)
Sepsis	37/102 (36.3%)

**Figure 1:**
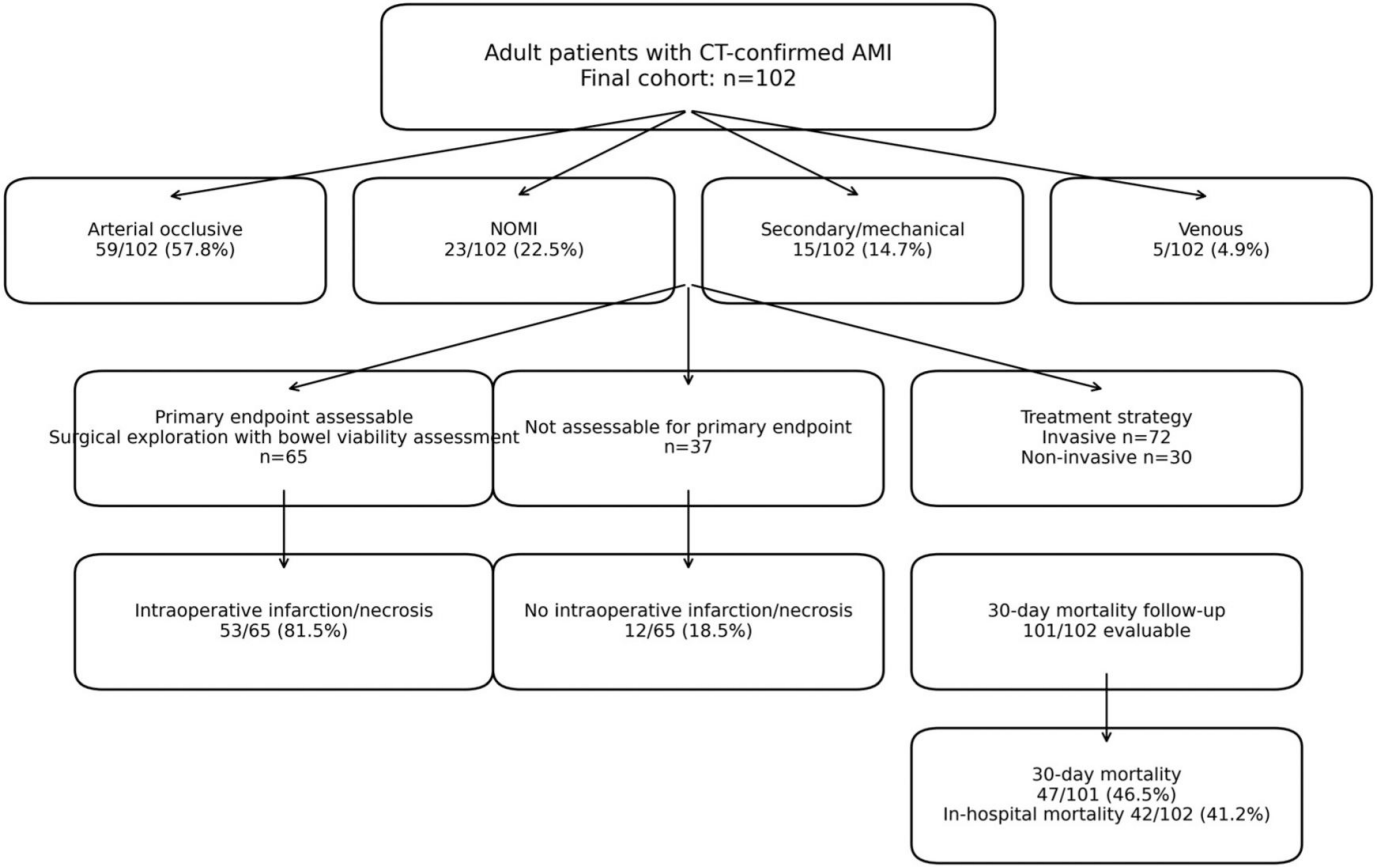
Study flow diagram. Flow diagram showing the final study cohort, AMI subtype distribution, availability of the primary intraoperative endpoint, treatment classification, and mortality follow-up.

Invasive treatment was performed in 72 patients (70.6%). Surgery was performed in 64 patients (62.7%), whereas interventional radiology or endovascular treatment was performed in 10 patients (9.8%). Two patients underwent combined surgical and interventional treatment. Bowel resection was documented in 36 of the 64 surgically evaluable patients (56.2%). Acute kidney injury was present in 23 patients (22.5%), diabetes mellitus in 16 (15.7%), pulmonary disease in 30 (29.4%), cardiac disease in 36 (35.3%), neurological disease in 10 (9.8%), oncohematologic disease in 21 (20.6%), and sepsis in 37 (36.3%). Initial admission to a non-surgical department occurred in 54 patients (52.9%), whereas 48 patients (47.1%) were initially admitted to a surgical department. Overall in-hospital mortality was 41.2% (42/102). Thirty-day mortality was 46.5% (47/101), including five patients who died after discharge within 30 days. One patient was not assessable for 30-day mortality (Figure 1).

### Radiological findings and exploratory CT ischemic burden score

The most frequent CT finding was vascular suffering or bowel wall hypoenhancement, present in 58 patients (56.9%), followed by pneumatosis intestinalis in 55 patients (53.9%) and free peritoneal fluid in 34 patients (33.3%). Bowel wall thickening was present in 21 patients (20.6%), occlusion/volvulus/intussusception in 20 patients (19.6%), porto-mesenteric venous gas in 13 patients (12.7%), free intraperitoneal air in 9 patients (8.8%), and colitis or ileitis in 6 patients (5.9%) (Table 2).

**Table 2:** Distribution of predefined CT findings, radiological diagnostic certainty, and exploratory CT ischemic burden score.

Radiological variable	*n* (%) or summary
Pneumatosis intestinalis	55/102 (53.9%)
Porto-mesenteric venous gas	13/102 (12.7%)
Bowel wall thickening	21/102 (20.6%)
Colitis/ileitis	6/102 (5.9%)
Occlusion/volvulus/intussusception	20/102 (19.6%)
Free intraperitoneal air	9/102 (8.8%)
Free peritoneal fluid	34/102 (33.3%)
Vascular suffering/hypoenhancement	58/102 (56.9%)
Radiological certainty (definite/probable)	84/102 (82.4%)
CT ischemic burden score (0–8), mean ± SD	2.1 ± 1.1
CT ischemic burden score (0–8), median (IQR)	2 (1–3)

Radiological diagnostic certainty, defined as definite or highly probable ischemia, was present in 84 patients (82.4%). Eighteen patients (17.6%) were classified as having unlikely or doubtful radiological ischemia. The exploratory CT ischemic burden score was calculated as the sum of the eight predefined CT findings. The score was analyzed as a descriptive measure of cumulative radiological abnormality burden and was not considered a validated prognostic or triage tool.

### Primary endpoint: intraoperative infarction or necrosis

The primary endpoint, intraoperative evidence of bowel infarction, necrosis, or irreversible ischemia, was assessable in 65 patients. Among these, 53 patients (81.5%) had intraoperative infarction or necrosis, whereas 12 patients (18.5%) had no intraoperative evidence of irreversible ischemic injury. Thirty-seven patients were not assessable for the primary endpoint because intraoperative evaluation of bowel viability was not available. The high proportion of positive intraoperative findings among evaluable patients reflects the severity of the surgically explored subgroup. However, the relatively small number of patients without infarction or necrosis limited statistical power and widened confidence intervals for the primary endpoint analysis.

### Association between radiological variables and intraoperative infarction or necrosis

All eight predefined CT findings and radiological diagnostic certainty were evaluated for their association with intraoperative infarction or necrosis (Table 3).

**Table 3:** Association between radiological variables and intraoperative infarction or necrosis. The table reports OR, 95% CI, *p*-value, sensitivity, specificity, PPV, NPV, AUC, and phi coefficient.

Radiological variable	*n*	events	OR (95% CI)	*p*-value	Sensitivity	Specificity	PPV	NPV	AUC	Phi coefficient
Pneumatosis intestinalis	65	53	2.72 (0.76–9.80)	0.188	0.66	0.58	0.88	0.28	0.62	0.19
Porto-mesenteric venous gas	65	53	0.89 (0.16–4.84)	1.000	0.15	0.83	0.80	0.18	0.49	−0.02
Bowel wall thickening	65	53	0.47 (0.12–1.86)	0.271	0.19	0.67	0.71	0.16	0.43	−0.14
Colitis/ileitis	65	53	1.21 (0.05–26.90)	1.000	0.04	1.00	1.00	0.19	0.52	0.08
Occlusion/volvulus/intussusception	65	53	1.08 (0.25–4.56)	1.000	0.26	0.75	0.82	0.19	0.51	0.01
Free intraperitoneal air	65	53	1.67 (0.19–15.05)	1.000	0.13	0.92	0.88	0.19	0.52	0.06
Free peritoneal fluid	65	53	0.26 (0.07–0.97)	0.052	0.34	0.33	0.69	0.10	0.34	−0.26
Vascular suffering/hypoenhancement	65	53	0.96 (0.28–3.37)	1.000	0.49	0.50	0.81	0.18	0.50	−0.01
Radiological certainty (definite/probable)	65	53	0.76 (0.15–4.00)	1.000	0.79	0.17	0.81	0.15	0.48	−0.04

No individual CT finding showed a statistically significant association with the primary endpoint. Pneumatosis intestinalis showed the strongest descriptive performance among the individual CT signs. Pneumatosis had an OR of 2.72 (95% CI: 0.76–9.80; *p* = 0.188), sensitivity of 66.0%, specificity of 58.3%, and AUC of 0.62. These findings suggest that pneumatosis was more frequent among patients with intraoperative infarction or necrosis, but it did not provide sufficient standalone discrimination.

Porto-mesenteric venous gas was infrequent and showed low sensitivity but relatively high specificity. Sensitivity was 15.1%, specificity was 83.3%, and AUC was 0.49. Free intraperitoneal air showed a similar pattern, with sensitivity of 13.2%, specificity of 91.7%, and AUC of 0.52. These findings indicate that porto-mesenteric venous gas and free air may support suspicion of advanced ischemic injury when present, but their absence cannot exclude infarction or necrosis.

Vascular suffering or bowel wall hypoenhancement had sensitivity of 49.1%, specificity of 50.0%, and AUC of 0.50. Occlusion/volvulus/intussusception showed sensitivity of 26.4%, specificity of 75.0%, and AUC of 0.51. Bowel wall thickening, colitis/ileitis, and free peritoneal fluid showed limited standalone discriminatory performance. Radiological diagnostic certainty showed high sensitivity but low specificity for intraoperative infarction or necrosis. This finding suggests that overall CT interpretation was sensitive for severe ischemic injury, but not sufficiently specific to identify irreversible infarction or necrosis when used alone.

Overall, the AUC values for individual CT findings were modest. These results indicate that no single radiological feature reliably discriminated patients with intraoperative infarction or necrosis from those without irreversible ischemic injury. CT findings therefore need to be interpreted in combination with AMI subtype, clinical severity, hemodynamic status, and surgical assessment.

### Secondary outcomes: clinically significant ischemia, bowel resection, and invasive treatment

Clinically significant ischemia was present in 75 patients (73.5%). This broader endpoint included intraoperative infarction or necrosis, bowel resection for ischemia or necrosis, histological confirmation when available, and non-operated patients with strong clinical-radiological evidence of irreversible ischemia.

In contrast to the primary intraoperative endpoint, pneumatosis intestinalis showed a stronger association with clinically significant ischemia. This supports the interpretation that pneumatosis may be more closely associated with a broader severe ischemic phenotype than with surgically verified infarction or necrosis alone. Porto-mesenteric venous gas and free intraperitoneal air remained relatively specific but infrequent findings.

Bowel resection was evaluated among surgically assessable patients. Resection was performed in 36 of 64 patients (56.2%). Because bowel resection may be influenced by disease extent, surgical strategy, technical feasibility, patient frailty, and operative judgment, it was considered a therapeutic endpoint rather than a pure diagnostic endpoint. Invasive treatment was performed in 72 patients (70.6%). This included surgical treatment, interventional radiology/endovascular treatment, or combined treatment. Associations between CT findings and invasive treatment were considered descriptive, because radiological findings directly influence treatment allocation and because treatment selection was not randomized.

### Thirty-day mortality and radiological findings

Thirty-day mortality was 46.5% among evaluable patients (47/101). Compared with in-hospital mortality, 30-day mortality identified five additional deaths occurring after discharge within 30 days. Among the radiological variables, pneumatosis intestinalis showed an exploratory univariate association with 30-day mortality. Patients with pneumatosis had higher odds of death within 30 days, with an OR of 2.42 (95% CI: 1.08–5.43; *p* = 0.045). Pneumatosis showed sensitivity of 66.0%, specificity of 55.6%, and AUC of 0.61 for 30-day mortality.

Porto-mesenteric venous gas was not significantly associated with 30-day mortality, although it showed high specificity and low sensitivity. Its OR was 2.01, with specificity of 90.7% and sensitivity of 17.0%.

Free intraperitoneal air showed a similar pattern of low prevalence and limited sensitivity.

Radiological diagnostic certainty showed high sensitivity but limited specificity for 30-day mortality. This indicates that definite or probable radiological ischemia was common among patients who died, but also common among survivors, limiting its discriminatory value as a prognostic marker of mortality.

The exploratory CT ischemic burden score showed limited performance for 30-day mortality. Each one-point increase in the score was not significantly associated with 30-day death, and the AUC was modest. These findings suggest that a simple additive count of CT abnormalities does not adequately capture mortality risk in this heterogeneous AMI population (Table 4).

**Table 4:** Association between radiological variables and 30-day mortality.

Radiological variable	*n*	events	OR (95% CI)	*p*-value	Sensitivity	Specificity	PPV	NPV	AUC	Phi coefficient
Pneumatosis intestinalis	101	47	2.42 (1.08–5.43)	0.045	0.66	0.56	0.56	0.65	0.61	0.22
Porto-mesenteric venous gas	101	47	2.01 (0.61–6.63)	0.372	0.17	0.91	0.62	0.56	0.54	0.12
Bowel wall thickening	101	47	0.50 (0.18–1.37)	0.222	0.15	0.74	0.33	0.50	0.44	−0.14
Colitis/ileitis	101	47	0.56 (0.10–3.18)	0.683	0.04	0.93	0.33	0.53	0.48	−0.07
Occlusion/volvulus/intussusception	101	47	0.31 (0.10–0.93)	0.044	0.11	0.72	0.25	0.48	0.41	−0.21
Free intraperitoneal air	101	47	0.30 (0.06–1.51)	0.170	0.04	0.87	0.22	0.51	0.46	−0.15
Free peritoneal fluid	101	47	0.50 (0.21–1.17)	0.140	0.26	0.59	0.35	0.48	0.42	−0.16
Vascular suffering/hypoenhancement	101	47	1.27 (0.58–2.80)	0.688	0.60	0.46	0.49	0.57	0.53	0.06
Radiological certainty (definite/probable)	101	47	1.46 (0.52–4.14)	0.604	0.85	0.20	0.48	0.61	0.53	0.07

The inverse association observed for occlusion/volvulus/intussusception and 30-day mortality should be interpreted cautiously, as this grouped variable includes mechanical or obstructive conditions that may differ substantially from NOMI or diffuse low-flow ischemia in terms of reversibility, surgical management, and prognosis.

### Mortality predictors and multivariable analysis

Clinical variables and comorbidities were first evaluated in univariate analysis for their association with mortality. These included age, sex, non-surgical admission, acute kidney injury, diabetes mellitus, pulmonary disease, cardiac disease, neurological disease, oncohematologic disease, and sepsis.

**Figure 2:**
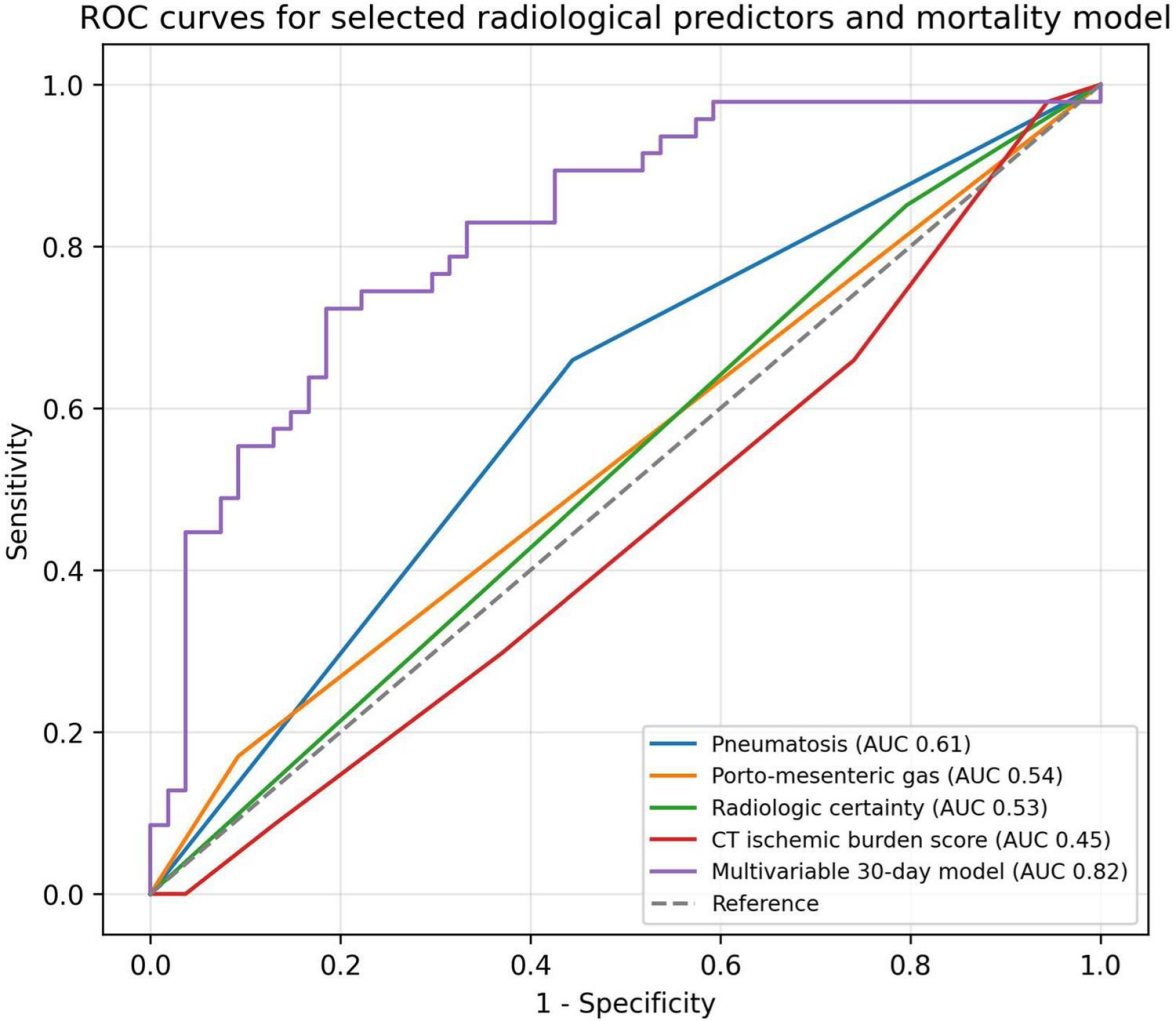
ROC curves for selected radiological predictors and mortality models. ROC curves are shown for selected CT variables, radiological diagnostic certainty, exploratory CT ischemic burden score, and the multivariable model for 30-day mortality.

A multivariable logistic regression model was then constructed for 30-day mortality using clinically relevant variables selected *a priori*. The final model included age, non-surgical admission, acute kidney injury, sepsis, and pulmonary disease. This model included 101 evaluable patients and showed good discrimination, with an AUC of 0.82. The ROC curves for selected radiological predictors and the multivariable 30-day mortality model are shown in Figure 2. In this model, acute kidney injury was independently associated with 30-day mortality, with an adjusted OR of 9.26 (95% CI: 2.40–35.74; *p* = 0.001). Initial admission to a non-surgical department showed a borderline association with 30-day mortality after adjustment, with an adjusted OR of 2.70 (95% CI: 0.94–7.71; *p* = 0.065). Age (adjusted OR: 1.02, 95% CI: 0.98–1.05; *p* = 0.309), sepsis (adjusted OR: 2.25, 95% CI: 0.82–6.16; *p* = 0.113), and pulmonary disease (adjusted OR: 1.43, 95% CI: 0.48–4.29; *p* = 0.526) were not independently associated with 30-day mortality.

A secondary multivariable model was performed for in-hospital mortality. This model included all 102 patients and also showed good discrimination, with an AUC of 0.82. In this model, initial admission to a non-surgical department was independently associated with in-hospital mortality (adjusted OR: 3.42, 95% CI: 1.18–9.93; *p* = 0.024). Acute kidney injury also remained independently associated with in-hospital mortality (adjusted OR: 6.67, 95% CI: 2.05–21.63; *p* = 0.002), as did sepsis (adjusted OR: 2.73, 95% CI: 1.02–7.30; *p* = 0.046). Age (adjusted OR: 1.01, 95% CI: 0.98–1.04; *p* = 0.662) and pulmonary disease (adjusted OR: 0.65, 95% CI: 0.21–1.97; *p* = 0.447) were not independently associated with in-hospital mortality (Table 5).

**Table 5:** Main multivariable logistic regression models for 30-day and in-hospital mortality.

Variable	Outcome	*N*	Events	Model AUC	Adjusted OR	95% CI	*p*-value
Age	30-day mortality	101	47	0.82	1.02	0.98–1.05	0.309
Non-surgical admission	30-day mortality	101	47	0.82	2.70	0.94–7.71	0.065
AKI/IRA	30-day mortality	101	47	0.82	9.26	2.40–35.74	0.001
Sepsis	30-day mortality	101	47	0.82	2.25	0.82–6.16	0.113
Pulmonary disease	30-day mortality	101	47	0.82	1.43	0.48–4.29	0.526
Age	In-hospital mortality	102	42	0.82	1.01	0.98–1.04	0.662
Non-surgical admission	In-hospital mortality	102	42	0.82	3.42	1.18–9.93	0.024
AKI/IRA	In-hospital mortality	102	42	0.82	6.67	2.05–21.63	0.002
Sepsis	In-hospital mortality	102	42	0.82	2.73	1.02–7.30	0.046
Pulmonary disease	In-hospital mortality	102	42	0.82	0.65	0.21–1.97	0.447

**Figure 3:**
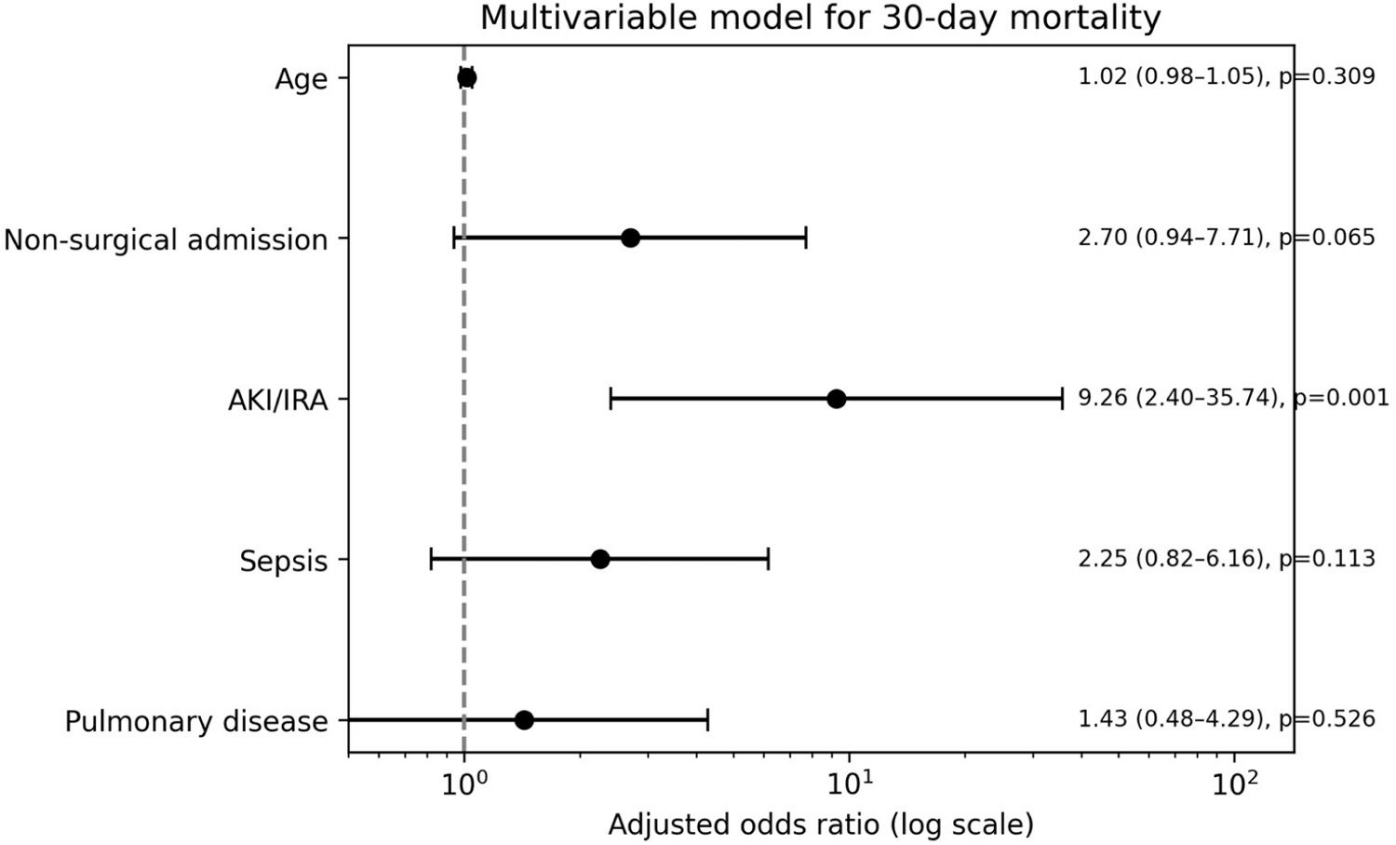
Forest plot of the multivariable logistic regression model for 30-day mortality. The forest plot shows adjusted odds ratios and 95% confidence intervals for the main multivariable model evaluating predictors of 30-day mortality. Acute kidney injury was independently associated with 30-day mortality, while initial admission to a non-surgical department showed a borderline association after adjustment. AKI, acute kidney injury; OR, odds ratio.

These findings indicate that acute kidney injury was the most consistent independent predictor of mortality across both models. Initial admission to a non-surgical department was independently associated with in-hospital mortality and showed a borderline association with 30-day mortality. Sepsis was independently associated with in-hospital mortality but not with 30-day mortality after adjustment (Figure 3).

### Treatment strategy and exploratory outcomes

Treatment strategy was analyzed descriptively. Thirty patients did not undergo invasive treatment. Sixty-two patients underwent surgery only, eight underwent interventional radiology or endovascular treatment only, and two underwent combined surgical and interventional treatment. Because treatment allocation was strongly influenced by AMI subtype, radiological findings, clinical severity, anatomical feasibility, operative risk, and perceived bowel viability, comparisons between treatment groups were considered exploratory. The study was not designed or powered to evaluate the independent efficacy of surgical or endovascular treatment. Interventional radiology/endovascular treatment was performed in a small selected subgroup and was most relevant in patients with arterial occlusive AMI. Given the limited number of patients treated with interventional radiology and the high risk of selection bias, no causal conclusions regarding reduction in bowel resection or mortality can be drawn from this subgroup.

## Discussion

Acute mesenteric ischemia remains a high-mortality emergency in which delayed diagnosis and progression from reversible ischemia to transmural necrosis strongly influence treatment options and prognosis (1–3). Contrast-enhanced CT is central to diagnosis and therapeutic planning. However, the prognostic interpretation of individual CT signs remains challenging because AMI includes heterogeneous entities with different mechanisms, imaging patterns, and clinical trajectories (4–7).

From an etiopathogenetic perspective, AMI includes arterial occlusive forms of embolic or thrombotic origin, venous mesenteric ischemia, non-occlusive mesenteric ischemia associated with systemic hypoperfusion and splanchnic vasoconstriction, and ischemia secondary to mechanical obstruction such as volvulus or strangulated hernias (8, 9). Ischemic injury progresses along a temporal continuum, from potentially reversible mucosal damage to irreversible transmural necrosis, directly influencing treatment strategy and prognosis (10–12). The limited reliability of clinical presentation and circulating biomarkers further reinforces the central role of imaging in AMI assessment (13–17).

Several CT findings, including impaired bowel wall enhancement, pneumatosis intestinalis, porto-mesenteric venous gas, free intraperitoneal air, and peritoneal fluid, have been associated with advanced ischemic injury or poor outcome in previous studies (18–22). However, these signs do not have uniform diagnostic or prognostic meaning across all AMI subtypes and clinical contexts. Emerging imaging approaches such as dual-energy CT may improve the assessment of bowel wall enhancement, but their role in routine AMI prognostic stratification remains insufficiently validated (23, 24). A further challenge in the available literature is the heterogeneity of diagnostic definitions, endpoints, and radiological criteria used to identify irreversible ischemia (25).

In this context, the present retrospective single-center cohort study evaluated the association between predefined CT findings, AMI subtypes, intraoperative evidence of infarction or necrosis, treatment pathways, and mortality in a real-world cohort of patients with CT-confirmed AMI. The main finding was that individual CT findings, although clinically meaningful, showed limited standalone discriminatory performance for intraoperative infarction or necrosis.

Pneumatosis intestinalis showed the strongest descriptive performance for the primary endpoint, but did not reach statistical significance and had only modest AUC. Porto-mesenteric venous gas and free intraperitoneal air showed relatively high specificity but low sensitivity, indicating that these signs may support suspicion of advanced ischemic injury when present, but their absence cannot exclude irreversible bowel injury. These findings are clinically plausible. Pneumatosis intestinalis and porto-mesenteric venous gas are widely recognized as important CT findings in intestinal ischemia, particularly when associated with impaired bowel wall enhancement, systemic deterioration, metabolic acidosis, peritonitis, or shock. However, these signs are not synonymous with transmural necrosis in every patient. Pneumatosis may be observed in different degrees of bowel wall injury and may occur in both transmural and non-transmural ischemia. Similarly, porto-mesenteric venous gas is an alarming sign, but its prognostic meaning depends on the underlying clinical setting, AMI subtype, and associated CT abnormalities.

The choice of endpoint likely influenced the observed performance of the CT findings. Intraoperative infarction or necrosis is a strong endpoint, but it is available only in surgically assessed patients. Patients with severe radiological findings who were not operated on because of advanced disease, prohibitive clinical status, or rapid deterioration are not captured by this endpoint. This may partly explain why pneumatosis showed a stronger association with clinically significant ischemia and with 30-day mortality than with the narrower intraoperative endpoint alone.

The exploratory CT ischemic burden score did not demonstrate robust discriminatory performance. This finding should not be interpreted as a limitation of CT itself, but rather as a limitation of a simple additive scoring approach. AMI is pathophysiologically heterogeneous. Arterial occlusive AMI, venous AMI, NOMI, and secondary/mechanical ischemia produce different imaging patterns, evolve through different mechanisms, and lead to different treatment pathways. A simple unweighted score assigning equal importance to all CT signs may therefore be insufficient to predict irreversible ischemia or mortality (25).

The mortality analysis provided clinically relevant information. Thirty-day mortality was higher than in-hospital mortality because it captured additional deaths occurring after discharge. Pneumatosis showed an exploratory univariate association with 30-day mortality, supporting its potential role as a marker of clinically severe disease. However, in the adjusted mortality models, systemic clinical severity appeared to be the dominant prognostic factor. Acute kidney injury was strongly associated with both 30-day and in-hospital mortality. Initial admission to a non-surgical department also showed a borderline association after adjustment.

The exploratory association between pneumatosis and 30-day mortality should be interpreted cautiously, particularly because multiple radiological variables were evaluated across several outcomes without formal adjustment for multiplicity. Therefore, this finding should not be considered confirmatory or sufficient for clinical decision-making in isolation. However, the association is biologically plausible, consistent with the known relationship between pneumatosis and advanced intestinal ischemic injury, and may support further investigation in independent prospective cohorts.

The association between non-surgical admission and mortality should not be interpreted as causal. Patients initially admitted to non-surgical units may have had atypical presentations, greater systemic illness, respiratory or cardiovascular instability, renal dysfunction, or diagnostic uncertainty. Nevertheless, this finding supports the clinical importance of early multidisciplinary assessment in patients with suspected AMI, particularly when abdominal symptoms occur in association with systemic deterioration.

Treatment strategy was analyzed descriptively because allocation to conservative, surgical, interventional, or combined treatment was strongly influenced by disease mechanism, radiological findings, vascular anatomy, clinical condition, and perceived operative feasibility. Interventional radiology/endovascular treatment was used in a small, selected subgroup, mainly relevant to arterial occlusive AMI. The present study was not powered to evaluate the independent efficacy of endovascular treatment, and no causal inference should be drawn from treatment comparisons. These findings support the concept that AMI should be managed as a time-dependent emergency requiring early multidisciplinary assessment. Although indocyanine green fluorescence angiography is available at our institution as a potential intraoperative adjunct for bowel viability assessment, this aspect was not systematically investigated in the present study and was not included in the statistical analysis (26–28).

Overall, the findings support an integrated approach to AMI. CT remains essential for diagnosis, subtype classification, and therapeutic planning. However, individual CT signs and simple additive scores should not be interpreted in isolation. Radiological findings must be integrated with AMI subtype, clinical severity, laboratory abnormalities, hemodynamic status, comorbidities, and early surgical assessment (29).

### Limitations

This study has several limitations. First, its retrospective single-center design limits generalizability and exposes the analysis to selection bias. Diagnostic pathways, availability of surgical and endovascular expertise, timing of CT, and institutional thresholds for operative exploration may vary across centers.

Second, AMI is a heterogeneous condition. Although AMI subtype was recorded and reported, the sample size was insufficient to perform robust subtype-specific predictive models. As a result, some associations may have been diluted by combining arterial, venous, non-occlusive, and secondary/mechanical forms in the same global analyses.

Third, the primary endpoint was based on intraoperative evidence of infarction, necrosis, or irreversible ischemia. This endpoint is clinically robust and avoids radiological incorporation bias, but it was assessable only in surgically evaluated patients. Non-operated patients were therefore excluded from the primary endpoint analysis, which may have introduced verification bias.

Fourth, the number of patients without intraoperative infarction or necrosis was small. This limited statistical power, widened confidence intervals, and restricted the complexity of multivariable models. For this reason, multivariable analyses were deliberately parsimonious and should be interpreted as exploratory. Variable selection for the multivariable models was based on clinical relevance rather than automated stepwise procedures or univariable *p*-value thresholds, and the models should therefore be interpreted as exploratory adjustment models rather than definitive prediction models.

Fifth, this study was exploratory and hypothesis-generating in design. Multiple statistical comparisons were performed without formal adjustment for multiplicity, increasing the risk of false-positive findings. Therefore, associations with borderline or modest statistical significance, including the association between pneumatosis and 30-day mortality, should be interpreted cautiously and require validation in independent prospective cohorts.

Sixth, several CT findings had low prevalence, particularly porto-mesenteric venous gas, free intraperitoneal air, and colitis/ileitis. Low prevalence reduces the ability to detect significant associations even when findings are clinically meaningful. Therefore, non-significant results should not be interpreted as evidence of clinical irrelevance.

Seventh, the exploratory CT ischemic burden score was not externally validated. It was designed as a simple unweighted sum of eight CT findings and should be considered a descriptive measure of radiological burden rather than a validated prognostic or triage tool.

Eighth, treatment decisions were influenced by clinical judgment, patient frailty, hemodynamic instability, comorbidities, institutional pathways, and surgical assessment. Therefore, associations between CT findings, treatment strategies, admission department, and outcomes cannot be interpreted causally.

Finally, although 30-day mortality was available for nearly all patients, detailed time-to-diagnosis and time-to-treatment variables were not available. Therefore, no conclusions can be drawn regarding the impact of diagnostic delay, timing of surgery, or timing of endovascular treatment on outcomes.

## Conclusions

In this real-world cohort of patients with CT-confirmed AMI, individual CT findings showed limited standalone discriminatory performance for intraoperative infarction or necrosis. Pneumatosis intestinalis showed the strongest descriptive association with the primary endpoint and an exploratory association with 30-day mortality, requiring validation in independent cohorts. Porto-mesenteric venous gas and free intraperitoneal air were specific but infrequent findings, with low sensitivity. The exploratory CT ischemic burden score, constructed as the sum of eight predefined CT findings, showed modest performance and should be interpreted as a descriptive measure of radiological burden rather than a validated prognostic or triage tool. AMI subtype heterogeneity likely contributes to the limited performance of simple radiological prediction models. Thirty-day mortality remained high and was independently associated with acute kidney injury, whereas non-surgical admission showed a borderline association after adjustment. These findings highlight the importance of integrating CT findings with AMI subtype, systemic clinical severity, and multidisciplinary assessment. CT is indispensable in AMI management, but imaging signs should not replace clinical judgment or surgical evaluation.

## Data Availability

The data analyzed in this study is subject to the following licenses/restrictions: The dataset contains de-identified and anonymized data. Is not publicly available due to privacy restrictions. However, it may be made available from the corresponding author upon reasonable request. Requests to access these datasets should be directed to the corresponding author, Francesco Leonforte, at leonfortefrancesco1@gmail.com.
