# Supplementary material for: Computed tomography findings and surgical outcomes in acute mesenteric ischemia: a retrospective single-center cohort study
Source: Front Surg. 2026 Jun 24;13:1824037. doi: 10.3389/fsurg.2026.1824037 (PMC13341654; doi:10.3389/fsurg.2026.1824037)
Supplement: Supplementary file 1 [file Datasheet1.pdf]

Supplementary Tables and Figures

Supplementary material for: Computed Tomography Findings and Surgical Outcomes in Acute Mesenteric Ischemia.

General note on interpretation: All supplementary analyses should be interpreted as exploratory and hypothesis-generating. No formal correction for multiple comparisons was applied. P-values are therefore reported as descriptive measures of association and should not be interpreted as confirmatory hypothesis tests. Findings, including associations with borderline or modest statistical significance, require validation in independent prospective cohorts before informing clinical decision-making or treatment algorithms.

Summary of dataset

Supplementary Table S0. AMI subtype classification.

Item	Updated value
AMI subtype: arterial occlusive	59/102 (57.8%)
AMI subtype: NOMI	23/102 (22.5%)
AMI subtype: secondary/mechanical	15/102 (14.7%)
AMI subtype: venous	5/102 (4.9%)
Primary endpoint assessable	65/102 (63.7%)
Intraoperative infarction/necrosis	53/65 (81.5%)
30-day mortality	47/101 (46.5%)

Supplementary Table. Association between radiological variables and Intraoperative infarction/necrosis.

Radiological variable	n	events	OR (95% CI)	p-value	Sensitivity	Specificity	PPV	NPV	AUC	Phi coefficient
Pneumatosis intestinalis	65	53	2.72 (0.76–9.80)	0.188	0.66	0.58	0.88	0.28	0.62	0.19
Porto-mesenteric venous gas	65	53	0.89 (0.16–4.84)	1.000	0.15	0.83	0.80	0.18	0.49	-0.02
Bowel wall thickening	65	53	0.47 (0.12–1.86)	0.271	0.19	0.67	0.71	0.16	0.43	-0.14
Colitis/ileitis	65	53	1.21 (0.05–26.90)	1.000	0.04	1.00	1.00	0.19	0.52	0.08
Occlusion/volvulus/intussusception	65	53	1.08 (0.25–4.56)	1.000	0.26	0.75	0.82	0.19	0.51	0.01
Free intraperitoneal air	65	53	1.67 (0.19–15.05)	1.000	0.13	0.92	0.88	0.19	0.52	0.06
Free peritoneal fluid	65	53	0.26 (0.07–0.97)	0.052	0.34	0.33	0.69	0.10	0.34	-0.26
Vascular suffering/hypoenhancement	65	53	0.96 (0.28–3.37)	1.000	0.49	0.50	0.81	0.18	0.50	-0.01
Radiological certainty (definite/probable)	65	53	0.76 (0.15–4.00)	1.000	0.79	0.17	0.81	0.15	0.48	-0.04

Supplementary Table. Association between radiological variables and Invasive treatment.

Radiological variable	n	events	OR (95% CI)	p-value	Sensitivity	Specificity	PPV	NPV	AUC	Phi coefficient
Pneumatosis intestinalis	102	72	1.83 (0.77–4.33)	0.195	0.58	0.57	0.76	0.36	0.58	0.14
Porto-mesenteric venous gas	102	72	2.52 (0.52–12.16)	0.335	0.15	0.93	0.85	0.31	0.54	0.12
Bowel wall thickening	102	72	0.79 (0.28–2.22)	0.789	0.19	0.77	0.67	0.28	0.48	-0.04
Colitis/ileitis	102	72	0.19 (0.03–1.08)	0.060	0.03	0.87	0.33	0.27	0.45	-0.20
Occlusion/volvulus/intussusception	102	72	4.67 (1.01–21.56)	0.053	0.25	0.93	0.90	0.34	0.59	0.21
Free intraperitoneal air	102	72	3.62 (0.43–30.34)	0.276	0.11	0.97	0.89	0.31	0.54	0.12
Free peritoneal fluid	102	72	1.55 (0.61–3.98)	0.490	0.36	0.73	0.76	0.32	0.55	0.09
Vascular suffering/hypoenhancement	102	72	0.56 (0.23–1.36)	0.273	0.53	0.33	0.66	0.23	0.43	-0.13
Radiological certainty (definite/probable)	102	72	0.91 (0.29–2.82)	1.000	0.82	0.17	0.70	0.28	0.49	-0.02

Supplementary Table. Association between radiological variables and Clinically significant ischemia.

Radiological variable	n	events	OR (95% CI)	p-value	Sensitivity	Specificity	PPV	NPV	AUC	Phi coefficient
Pneumatosis intestinalis	102	75	2.55 (1.03–6.32)	0.046	0.60	0.63	0.82	0.36	0.61	0.20
Porto-mesenteric venous gas	102	75	0.78 (0.22–2.79)	0.741	0.12	0.85	0.69	0.26	0.49	-0.04
Bowel wall thickening	102	75	0.38 (0.14–1.05)	0.093	0.16	0.67	0.57	0.22	0.41	-0.19
Colitis/ileitis	102	75	0.16 (0.03–0.92)	0.041	0.03	0.85	0.33	0.24	0.44	-0.23
Occlusion/volvulus/intussusception	102	75	2.34 (0.63–8.75)	0.263	0.23	0.89	0.85	0.29	0.56	0.13
Free intraperitoneal air	102	75	3.10 (0.37–26.06)	0.439	0.11	0.96	0.89	0.28	0.53	0.11
Free peritoneal fluid	102	75	0.64 (0.26–1.60)	0.352	0.31	0.59	0.68	0.24	0.45	-0.09
Vascular suffering/hypoenhancement	102	75	0.71 (0.29–1.75)	0.503	0.55	0.37	0.71	0.23	0.46	-0.07
Radiological certainty (definite/probable)	102	75	1.08 (0.35–3.39)	1.000	0.83	0.19	0.74	0.28	0.51	0.01

Supplementary Table. Association between radiological variables and Bowel resection.

Radiological variable	n	events	OR (95% CI)	p-value	Sensitivity	Specificity	PPV	NPV	AUC	Phi coefficient
Pneumatosis intestinalis	64	36	1.14 (0.41–3.17)	0.801	0.64	0.39	0.57	0.46	0.52	0.03
Porto-mesenteric venous gas	64	36	2.01 (0.47–8.61)	0.492	0.19	0.89	0.70	0.46	0.54	0.12
Bowel wall thickening	64	36	1.31 (0.38–4.57)	0.761	0.22	0.82	0.62	0.45	0.52	0.05
Colitis/ileitis	64	36	4.13 (0.19–89.57)	0.500	0.06	1.00	1.00	0.45	0.53	0.16
Occlusion/volvulus/intussusception	64	36	1.61 (0.51–5.08)	0.570	0.31	0.79	0.65	0.47	0.55	0.10
Free intraperitoneal air	64	36	0.75 (0.17–3.31)	0.721	0.11	0.86	0.50	0.43	0.48	-0.05
Free peritoneal fluid	64	36	0.65 (0.24–1.79)	0.450	0.36	0.54	0.50	0.39	0.45	-0.10
Vascular suffering/hypoenhancement	64	36	1.49 (0.55–4.03)	0.461	0.53	0.57	0.61	0.48	0.55	0.10
Radiological certainty (definite/probable)	64	36	0.50 (0.14–1.83)	0.359	0.75	0.14	0.53	0.31	0.45	-0.13

Supplementary Table. Association between radiological variables and In-hospital mortality.

Radiological variable	n	events	OR (95% CI)	p-value	Sensitivity	Specificity	PPV	NPV	AUC	Phi coefficient
Pneumatosis intestinalis	102	42	2.06 (0.92–4.62)	0.107	0.64	0.53	0.49	0.68	0.59	0.17
Porto-mesenteric venous gas	102	42	1.26 (0.39–4.06)	0.767	0.14	0.88	0.46	0.60	0.51	0.04
Bowel wall thickening	102	42	0.50 (0.18–1.42)	0.221	0.14	0.75	0.29	0.56	0.45	-0.13
Colitis/ileitis	102	42	0.70 (0.12–4.01)	1.000	0.05	0.93	0.33	0.58	0.49	-0.04
Occlusion/volvulus/intussusception	102	42	0.29 (0.09–0.94)	0.042	0.10	0.73	0.20	0.54	0.41	-0.21
Free intraperitoneal air	102	42	0.38 (0.07–1.92)	0.301	0.05	0.88	0.22	0.57	0.47	-0.12
Free peritoneal fluid	102	42	0.47 (0.19–1.13)	0.135	0.24	0.60	0.29	0.53	0.42	-0.17
Vascular suffering/hypoenhancement	102	42	1.20 (0.54–2.68)	0.689	0.60	0.45	0.43	0.61	0.52	0.04
Radiological certainty (definite/probable)	102	42	1.12 (0.40–3.18)	1.000	0.83	0.18	0.42	0.61	0.51	0.02

Supplementary Table. Association between radiological variables and 30-day mortality.

Note: Associations in this table are exploratory. In particular, the association between pneumatosis intestinalis and 30-day mortality should be interpreted cautiously because multiple radiological variables were evaluated across several outcomes without formal multiplicity adjustment.

Radiological variable	n	events	OR (95% CI)	p-value	Sensitivity	Specificity	PPV	NPV	AUC	Phi coefficient
Pneumatosis intestinalis	101	47	2.42 (1.08–5.43)	0.045	0.66	0.56	0.56	0.65	0.61	0.22
Porto-mesenteric venous gas	101	47	2.01 (0.61–6.63)	0.372	0.17	0.91	0.62	0.56	0.54	0.12
Bowel wall thickening	101	47	0.50 (0.18–1.37)	0.222	0.15	0.74	0.33	0.50	0.44	-0.14
Colitis/ileitis	101	47	0.56 (0.10–3.18)	0.683	0.04	0.93	0.33	0.53	0.48	-0.07
Occlusion/volvulus/intussusception	101	47	0.31 (0.10–0.93)	0.044	0.11	0.72	0.25	0.48	0.41	-0.21
Free intraperitoneal air	101	47	0.30 (0.06–1.51)	0.170	0.04	0.87	0.22	0.51	0.46	-0.15
Free peritoneal fluid	101	47	0.50 (0.21–1.17)	0.140	0.26	0.59	0.35	0.48	0.42	-0.16
Vascular suffering/hypoenhancement	101	47	1.27 (0.58–2.80)	0.688	0.60	0.46	0.49	0.57	0.53	0.06
Radiological certainty (definite/probable)	101	47	1.46 (0.52–4.14)	0.604	0.85	0.20	0.48	0.61	0.53	0.07

Supplementary Table. Association between radiological variables and Non-surgical admission.

Radiological variable	n	events	OR (95% CI)	p-value	Sensitivity	Specificity	PPV	NPV	AUC	Phi coefficient
Pneumatosis intestinalis	102	54	1.58 (0.72–3.46)	0.320	0.59	0.52	0.58	0.53	0.56	0.11
Porto-mesenteric venous gas	102	54	0.51 (0.15–1.68)	0.374	0.09	0.83	0.38	0.45	0.46	-0.11
Bowel wall thickening	102	54	1.59 (0.59–4.24)	0.463	0.24	0.83	0.62	0.49	0.54	0.09
Colitis/ileitis	102	54	0.88 (0.17–4.59)	1.000	0.06	0.94	0.50	0.47	0.50	-0.01
Occlusion/volvulus/intussusception	102	54	0.30 (0.11–0.87)	0.026	0.11	0.71	0.30	0.41	0.41	-0.23
Free intraperitoneal air	102	54	0.41 (0.10–1.75)	0.300	0.06	0.88	0.33	0.45	0.47	-0.12
Free peritoneal fluid	102	54	0.49 (0.21–1.13)	0.099	0.26	0.58	0.41	0.41	0.42	-0.17
Vascular suffering/hypoenhancement	102	54	0.89 (0.41–1.96)	0.843	0.56	0.42	0.52	0.45	0.49	-0.03
Radiological certainty (definite/probable)	102	54	0.88 (0.32–2.45)	1.000	0.81	0.17	0.52	0.44	0.49	-0.02

Supplementary Table. Exploratory CT ischemic burden score across study outcomes.

Outcome	N	Events	OR per point	95% CI	p-value	AUC	Mean score events	Mean score non-events
Intraoperative infarction/necrosis	65	53	0.889	0.51-1.54	0.674	0.45	2.264	2.417
Invasive treatment	102	72	1.318	0.87-1.99	0.192	0.57	2.208	1.9
Clinically significant ischemia	102	75	0.924	0.62-1.39	0.704	0.46	2.093	2.185
Bowel resection	64	36	1.238	0.80-1.93	0.344	0.554	2.417	2.143
In-hospital mortality	102	42	0.781	0.54-1.14	0.199	0.428	1.952	2.233
30-day mortality	101	47	0.84	0.58-1.21	0.352	0.449	2.021	2.222
Non-surgical admission	102	54	0.75	0.52-1.09	0.128	0.427	1.963	2.292

Supplementary Table. Univariate clinical and comorbidity associations with mortality outcomes.

Outcome	Variable	N	Events	OR	95% CI	p-value
30-day mortality	Age	101	47	1.014	0.99-1.04	0.293
30-day mortality	Male sex	101	47	1.15	0.52-2.52	0.842
30-day mortality	Non-surgical admission	101	47	4.007	1.74-9.23	0.001
30-day mortality	AKI/IRA	101	47	12.593	3.43-46.21	<0.001
30-day mortality	Diabetes	101	47	1.59	0.54-4.67	0.426
30-day mortality	Pulmonary disease	101	47	2.986	1.21-7.35	0.027
30-day mortality	Cardiac disease	101	47	2.308	1.00-5.33	0.060
30-day mortality	Neurological disease	101	47	2.975	0.72-12.24	0.182
30-day mortality	Oncohematologic disease	101	47	0.926	0.35-2.47	1.000
30-day mortality	Sepsis	101	47	3.652	1.55-8.63	0.003
In-hospital mortality	Age	102	42	1.003	0.98-1.03	0.837
In-hospital mortality	Male sex	102	42	1.247	0.56-2.76	0.687
In-hospital mortality	Non-surgical admission	102	42	4.534	1.91-10.74	<0.001
In-hospital mortality	AKI/IRA	102	42	8.25	2.74-24.80	<0.001
In-hospital mortality	Diabetes	102	42	1.133	0.39-3.33	1.000
In-hospital mortality	Pulmonary disease	102	42	1.667	0.71-3.94	0.275
In-hospital mortality	Cardiac disease	102	42	1.231	0.54-2.80	0.676
In-hospital mortality	Neurological disease	102	42	3.8	0.92-15.67	0.087
In-hospital mortality	Oncohematologic disease	102	42	0.657	0.24-1.80	0.465
In-hospital mortality	Sepsis	102	42	3.977	1.70-9.33	0.002

Supplementary Table. Exploratory outcomes according to treatment strategy.

Treatment group	N	30-day mortality	In-hospital mortality	Bowel resection	Intraoperative infarction/necrosis	Clinically significant ischemia
No invasive treatment	30	15/29 (51.7%)	11/30 (36.7%)	NA	0/1 (0.0%)	16/30 (53.3%)
Surgery only	62	25/62 (40.3%)	24/62 (38.7%)	36/62 (58.1%)	52/62 (83.9%)	52/62 (83.9%)
Interventional radiology/endovascular only	8	6/8 (75.0%)	6/8 (75.0%)	NA	NA	5/8 (62.5%)
Combined surgical + interventional	2	1/2 (50.0%)	1/2 (50.0%)	0/2 (0.0%)	1/2 (50.0%)	2/2 (100.0%)

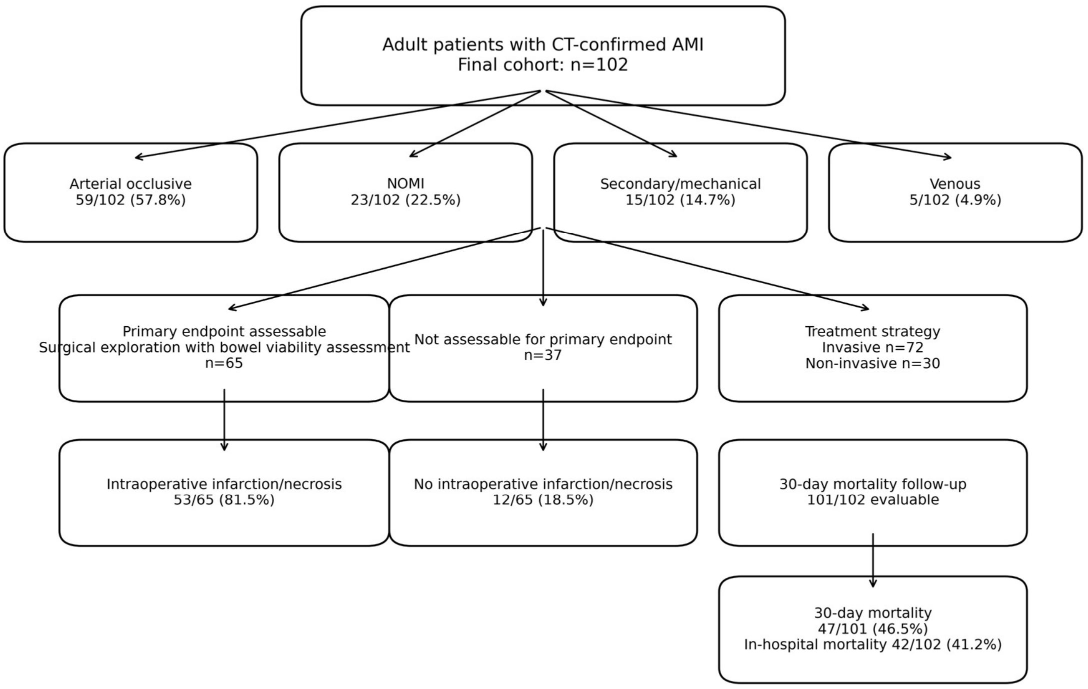

Supplementary Figure. Updated study flow diagram with revised AMI subtype distribution.

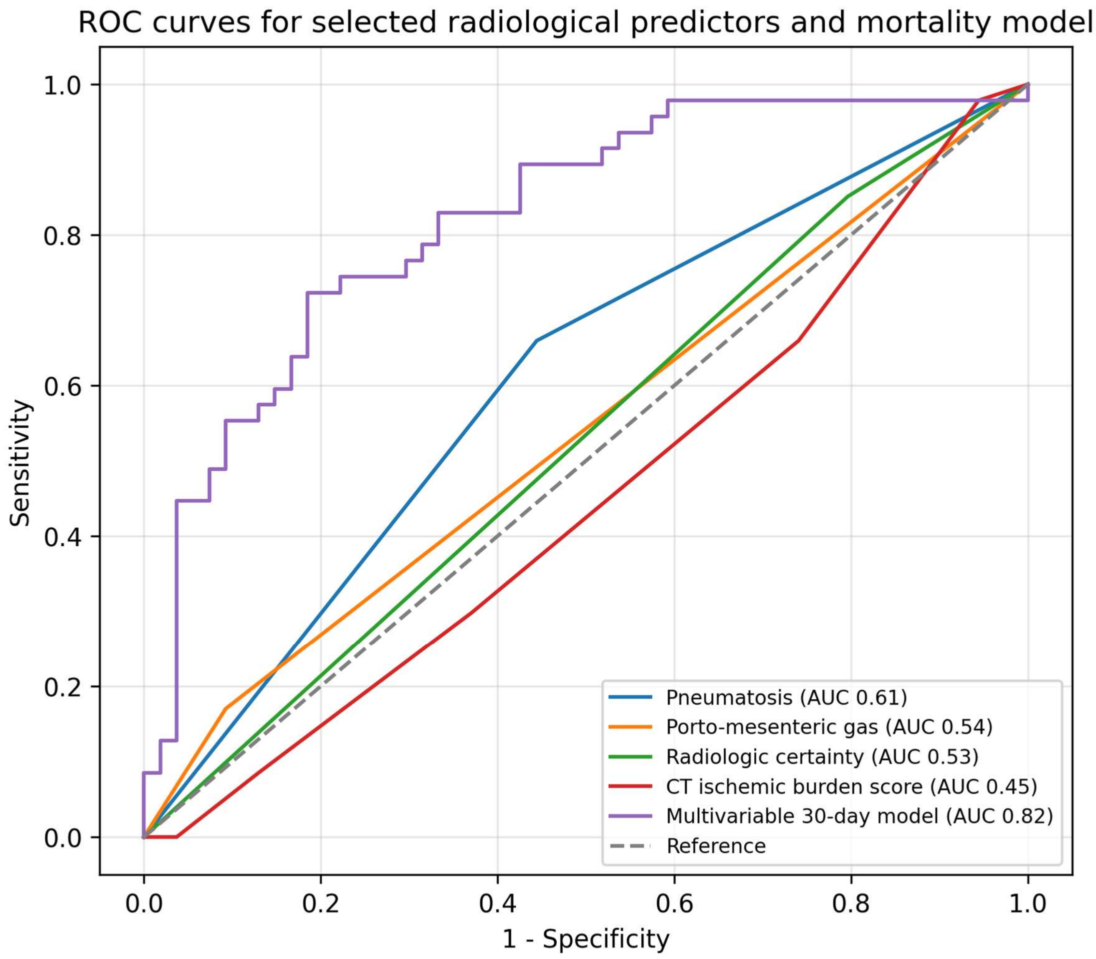

Supplementary Figure. Updated ROC curves.

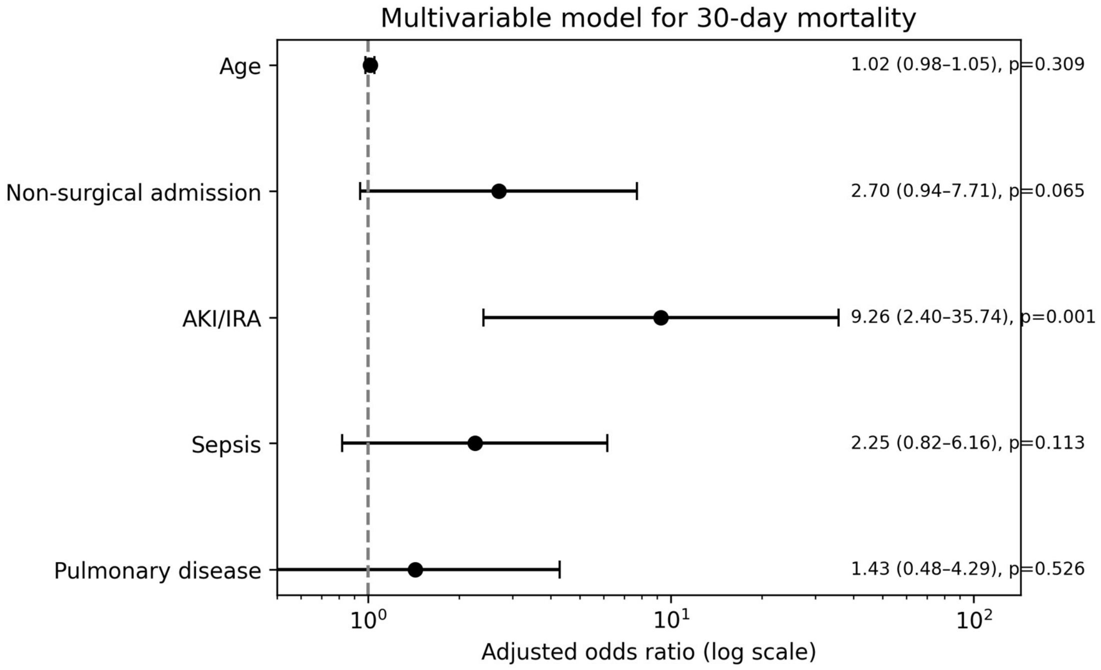

Supplementary Figure. Updated forest plot for the 30-day mortality model.
